# Physical Activity Reduces Depression among Healthcare Workers during the COVID-19 Pandemic in Jordan

**DOI:** 10.2174/17450179-v19-230720-2023-8

**Published:** 2023-07-25

**Authors:** Abdulhakeem Okour, Basil Amarneh

**Affiliations:** 1 Department of Public Health, Jordan University of Science and Technology, Amman, Jordan; 2 Nursing Community Mental Health, Jordan University of Science and Technology, Amman, Jordan

**Keywords:** COVID-19, SARS-CoV-2, Mental health issues, Physical activity, Patient, Depression

## Abstract

**Aims:**

This study aimed to assess mental health status by measuring depression and investigating the effect of physical activity in lessening the mental health burden among HCWs during the COVID-19 pandemic in Jordan.

**Background:**

The COVID-19 pandemic has caused distress and depression among healthcare workers and drastic disruptions in social, economic, and health systems worldwide.

**Methods:**

A cross-sectional online study through google forms involved HCWs across Jordan for two months. The 10-item short form of the Center for Epidemiological Studies Depression Scale Revised (CESD-R10) was used to assess depressive symptoms.

**Results:**

The total sample was 295, females were 50.5% with mean age of 33.1 (>80% less than 40 years old), married 51.9%, 63% were physicians, 88.1% had contact with patients, and 64.7% were smokers. Depression symptoms were perceived by 59.3% of the total samples (53.1).

**Conclusion:**

During the COVID-19-induced nationwide lockdown in Jordan, HCWs who engaged in enough physical exercise reported fewer depressive symptoms. Promoting physical exercise among HCWs may lead to better results for their mental health. Various clinical implications support promoting designated time and space for physical activity at work.

## 1 INTRODUCTION

The severe acute respiratory coronavirus 2 (SARS-CoV-2 or COVID-19), which emerged at the end of 2019, has caused a global pandemic [1], leading to profound fears and distress among healthcare workers (HCW) and drastic disruptions in social, economic, and health systems worldwide [1, 2]. HCWs are among the highest exposed people to contract COVID-19 due to its ability to spread so efficiently through asymptomatic and symptomatic individuals [3]. The scarcity of information concerning the risk of acquiring the infection, mode of transmission, and treatment has intensified the psychological burden experienced by HCWs during this unprecedented event. Furthermore, increasing cases, insufficient personal protection equipment (PPE), extremely demanding workloads, and lack of adequate therapies to save lives contributed to a burden directly related to working during disease outbreaks [1, 3].

The literature on HCWs working during epidemics and COVID-19 indicated increased mental burden among HCWs, including reported symptoms of post-traumatic stress, depression, anxiety, fear, and insomnia [4-6]. Perceived causes include social isolation, uncertainty around the future of the pandemic, availability of a vaccine, increased workload, lack of social support, and fear of familial transmission were all reported in more than one study [6-8]. Although psychosocial issues are common among HCWs, most mental health problems among health professionals stay hidden without seeking mental healthcare [8]. Depression is a common mental health condition that can majorly impact individual welfare and daily functioning [9]. According to the World Health Organization, depression affects around 322 million people and is ranked the most significant contributor to global disability (7.5% of all years lived with disability in 2015) [10]. Depression is characterized by persistent low mood, dysphoria, poor motivation, and several other symptoms, ranging from psychomotor to cognitive impairments. Depression can affect a person to suffer severely and malfunction at work, school, and family. In addition, it can cause severe physical health comorbidities, including cardiovascular disease, metabolic risk factors such as adiposity, and premature mortality. At its worst, depression can lead to suicide [9, 10]. Although effective treatments are available for mental disorders, more than 75% of people in low- and middle-income countries receive no treatment [9, 10]. Barriers to efficient care include lacking resources and/or trained healthcare providers and the social stigma associated with mental illnesses. Regardless of income level, people who experience depression are often not correctly diagnosed, and others who do not have the disorder are too often misdiagnosed and prescribed antidepressants [9].

Physical activity can protect against many morbidities, including mental health problems [11, 12]. The Centers for Disease Control and Prevention (CDC) and the American College of Sports Medicine (ACSM) recommend that adults exercise for at least 30 min on most days to improve their health and quality of life. Clinical trials have confirmed that regular exercise effectively treats the disease, including physical ailments, *e.g*., cardiovascular disease, and psychiatric disorders, *e.g*., depression. Further, cross-sectional studies frequently associate regular exercise with general well-being and lower mood and anxiety disorder rates [13, 14].

Jordan is a middle-income country in the middle east, where HCWs who confronted the COVID-19 pandemic were stationed at the Ministry of Health, army, universities, UNRWA, and private sector. By Sept 11, 2022, registered and confirmed cases of COVID-19 reached 1,738,867, and 14,114 deaths, a 7-day average of 482 COVID-19 cases [15]. Data on COVID-19 magnitude and the number of infected or dead HCWs in Jordan is limited. However, some studies on depression among HCWs in Jordan revealed significant findings [16-21]. Extremely severe depression (57.8%) among Jordanian HCWs and extremely severe anxiety (60%) were reported [16, 17]. Other studies indicated a moderate to high prevalence of depression ranging from 26% to 42% among HCWs in Jordan [18-21]. This study aimed to assess mental health status by measuring depression and investigating the effect of physical activity in lessening the mental health burden among HCWs during the COVID-19 pandemic in Jordan.

## 2 METHODOLOGY

This was a cross-sectional online study involving HCWs across Jordan for two months (from June to July 2021). Data was collected online through google forms, approaching and targeting 335 HCWs by their social networks groups and addresses, with a response rate of 88.3%. The study tool was developed to assess depressive symptoms using the 10-item short form of the Center for Epidemiological Studies Depression Scale Revised (CESD-R10), the reported Internal consistency for the CES-D-10 = (Cronbach's α=0.86) [20-24]. Participants answered each item of the scale and rated how frequently during the past 7 days and on a one to four Likert scale (0 = rarely- less than 1 day, 1 = some or a small quantity of the time- 1–2 days, 2 = occasionally or a moderate amount of time- 3–4 days, and 3 = most or all of the time- 5–7 days) they had experienced specific symptoms. The scale ranges from 0 to 30, and a score ≥ 10 denotes elevated depressive symptoms. A binary indicator was then created using a cutoff of 10 or more to indicate depressive symptoms. This cutoff is the most commonly used threshold and has been previously used in other studies [19-23]. Physical activity amount was defined in five categories (no physical activity, daily, weekly-one day, two days, 3 to 4 days), all for 30 min or less. For analysis, physical activity was reduced into two categories (1=no physical activity, 2=daily to weekly for 30 min). Sociodemographic variables included age, gender, specialty, marital status, patient contact, and smoking. The analyses included frequency distribution detailed by male-female, binary chi-square testing, and logistic regression at the alfa level equal to or less than 0.05. Jordan University of Science and Technology funded this study. The ethical authorization was granted by the Jordan University of Science & Technology Institutional Review Board (IRB); the approval required keeping data confidential and using it only for scientific purposes, providing a consent form, and providing the final research results report to the university. To meet the consent form requirement, the participant needed to click agree to start the online survey, and this agreement was considered the required consent to start filling out the scale.

## 3 RESULTS

The total sample participants were 295, and females were 50.5%, mean age was 33.1 (>80% less than 40 years old), married were 51.9%, 63% were physicians, 88.1% had contacts with patients, and 64.7% were smokers, (Table **1**). Depression symptoms were perceived by 59.3% of the total sample (53.1% females *Vs.* 46.9% males), while 61% were practicing physical activity daily or weekly for 30 min, (Table **2**). The relationship between depression status and sociodemographic and physical activity is shown in Table **3**. The results indicate a significant relationship between depression status and physical activity (p-value 0.003) and marital status (p-value 0.038), while all other factors were insignificant. Depression symptoms were mainly perceived among the younger age group (20-30 years old, 76.7%), females (53.1%), those not married (53.1%), and physicians (66.3%). Table **4** demonstrates the results of the binary regression analysis of depression status and other factors. Depression symptoms were more perceived among those who did not practice physical activity compared to those who did, P-value= 0.003, OR=2.18, 95% CI (1.30, 3.65), those who were smoking, P-value= 0.039, OR=0.543, 95%CI (0.303, 0.971), and females, P-value= 0.038, OR=0.551, 95%CI (0.313, 0.969).

## 4 DISCUSSION

The work performed by HCWs during ordinary times represents a risk that often cannot be avoided. Nevertheless, being at the frontlines confronting dangerous diseases during emergencies is an obligation for HCWs. This study positively promotes physical activity among HCWs to prevent depression symptoms, especially during tense situations. The results of this study established a significant relationship between the high level of perception of depression symptoms and lack of physical activity. This significant relationship was evident in the binary analysis and the logistic regression model (P-value= 0.003, OR= 2.18, 95% CI = (1.30, 3.65). This result agrees with the abundance of previous studies that confirmed the constructive function of physical activity in reducing the risk of deteriorating HCWs' mental health due to the pressure of the tremendous work performed [9-12]. Furthermore, physical activity part is shown as a protector against illness [9, 11, 13].

Almost 61% of the HCWs showed involvement in physical activities, which was associated with lower levels of depression compared to HCWs who did not involve in any physical activity.

These results validated our hypothesis and aligned with earlier studies among various demographics conducted during lockdowns. For instance, decreased weekly physical activity levels were negatively associated with well-being on the French Reunion Island and mood in England [24-28]. In the same way, Ernsten *et al.* reported that symptoms of anxiety and depression were considerably lower in physically active Norwegian adults [29].

The results of this study imply that HCWs may have dramatically reduced their levels of physical activity during the lockdown. In a Middle Eastern and North African country survey, Jordanian adults exhibited the highest pooled prevalence values of sufficient physical activity levels among girls and males (94.2% and 95.5%, respectively) [30]. However, it is essential to emphasize that comparing accurately with other conflicting literature reports is challenging due to varying definitions of physical activity.

For instance, another study published by Walke *et al.* reported that 51.8% of Jordanian adults did not engage in moderate physical activity [31]. Moreover, the results show that they mainly perceived depression symptoms among the younger age groups, females (53.1%), unmarried women, and physicians.

## 5 RECOMMENDATIONS

HCW populations should continue to be a focus of future research because they have received very little attention from previous studies on public health. In addition, there is a critical need for longitudinal research with accurate assessments of physical activity, depression, mental health, and studies using large cohorts. This allows researchers and policymakers to understand better the prevalence of poor rest and physical inactivity in these populations and the complex pathway(s) linking physical activity and mental health to inform public health policies and HCW initiatives. Furthermore, these behavioral and mental health characteristics affect immunity, the likelihood of developing chronic diseases, and suicidality, which is especially important during the current worldwide pandemic [32, 33]. Therefore, it is vital to consider intervention strategies that could stop the worsening of these health determinants and their subsequent impacts on health span and disease risk, especially for HCWs during lockdown times. This includes promoting transmission-safe pursuits like socially isolated outdoor solitude (*e.g*., walking and jogging). In addition, further thought should be given to the potential moderating effects of characteristics such as gender, life stage, pre-pandemic physical activity levels, and fitness levels.

In cases where poor mental health may constrict a person's motivation to exercise, it is also crucial to consider other interventions that directly improve mental health outcomes. One such intervention is supporting HCWs with mental health issues so they can engage in physical activity. Finally, qualitative research findings should be considered in future studies. Such research can help better understand the mechanism(s) behind the link between physical activity and various health outcomes, which can help inform intervention strategies and boost population health under lockdown situations.

## 6 STRENGTHS AND WEAKNESS

There were several significant advantages to this study. First, this is one of the few pieces of research on HCW populations that has examined the connection between health habits during a lockdown and risk factors for the most common chronic diseases. In addition, such actions have been connected to suicidality and immunity, two serious public health issues during pandemics.

Furthermore, the middle east and HCWs remain a region with little public health data and resources to fight the epidemic compared to Western countries. It is also a subject that receives less attention from public health researchers. Therefore, our study greatly expands public health knowledge about the HCW community. To quantify depression symptoms, this study used widely accepted and validated instruments.

This study had shortcomings despite its advantages. First off, this study used a cross-sectional design. Although it offers valuable information on correlations between the exposure and the desired result, temporality cannot be established, particularly for these likely bidirectional relationships, and causal inference cannot be made. In addition, social desirability and recollection bias might affect a self-reported survey, especially since not all HCWs have accounts with communication networks. Moreover, one of the shortcomings of using the social networking approach is that it did not reach every HCW, especially those not using it. Nevertheless, in terms of protecting participants' and researchers' safety during the COVID-19-induced lockdown, the online survey was the best approach that could be employed to gather information from a sizable sample.

## CONCLUSION

During the statewide lockdown brought on by COVID-19 in Jordan, HCWs who engaged in enough physical exercise reported fewer depressive symptoms, and HCWs' mental health outcomes might be improved by encouraging physical activity. The need for longitudinal population-based studies using established and impartial assessment technologies is required to separate these complicated connections and provide more solid evidence to inform public health policies and interventions

## Figures and Tables

**Table 1:** Sociodemographic characteristics by gender of participants.

-	**Female**	**Male**	**Total N**
**Age** 20-39 ≥ 40 Total	130 (54.6%) 19 (33.3%) 149 (50.5%)	108 (45.4%) 38 (66.7%) 146 (49.5%)	238 (80.7%) 57 (19.3%) 295 (100%)
**Marital status** Married Not married Total	85 (55.6%) 64 (45.1%)	68 (44.4%) 78 (54.9.%)	153 (51.9%) 142 (48.1%) 295 (100%)
**Specialty** Physicians Dentist Pharmacist Nurse Other Medical Total	68 (36.6%) 23 (67.6%) 28 (82.4%) 15 (68.2%) 15 (78.9%)	118 (63.4%) 11 (32.4%) 6 (17.6%) 7 (31.8%) 4 (21.1%)	186 (63.1%) 34 (11.5%) 34 (11.5%) 22 (7.5%) 19 (6.4%) 295 (100%)
**Contact with patients** Yes No Total	127 (48.8%) 22 (62.9%)	133 (51.2%) 13 (37.1%)	260 (88.1%) 35 (11.9%) 295 (100%)
**Smoking** Yes No Total	22 (21.2%) 127 (66.5%)	82 (78.8%) 64 (33.5%)	104 (64.7%) 191 (35.3%) 295 (100%)

**Table 2:** Depression status and physical activity of participants.

-	**Female**	**Male**	**Total N**
**Depression** Yes No	93 (53.1%) 56 (46.7%)	82 (46.9%) 64 (53.3%)	175 (59.3%) 120 (40.7%)
**Physical activity** Yes No	91 (50.3%) 58 (50.9%)	90 (49.7%) 56 (49.1%)	181 (61.4%) 114 (38.6%)
**Physical activity /yes** Daily for 30 min or less Daily for more than 30 min Weekly one day for 30 min Weekly two days for 30 min Weekly 3-4 days for 30 min	26 (46.4%) 5 (20.8%) 20 (52.6%) 22 (14.8%) 18 (12.1%)	30 (53.6%) 19 (79.2%) 18 (47.1%) 7 (4.8%) 16 (11%)	56 (19%) 24 (8.1%) 38 (12.9%) 29 (9.8%) 34 (11.5%)

**Table 3:** Binary relationship of depression status, physical activity of participants, and other factors.

-	**Depression**	**No Depression**	**p-value**
**Age** 20-39 ≥ 40	92 (76.7%) 28 (23.3%)	146 (83.4%) 29 (16.6%)	0.148
**Gender** Female Male	93 (53.1%) 82 (46.9%)	56 (46.7%) 64 (53.3%)	0.484
**Marital status**s Married Not married	71 (46.4%) 49 (34.5%)	82 (53.6%) 93 (65.5%)	0.038
**Specialty** Physicians Dentist Pharmacist Nurse Other Medical	70 (37.6%) 14 (41.2%) 15 (44.1%) 14 (63.6%) 7 (36.8%)	116 (62.4%) 20 (58.8%) 19 (55.9%) 8 (36.4%) 12 (63.2%)	0.214
**Contact with patients** Yes No Total	105 (40.4%) 15 (42.9%)	155 (59.6%) 20 (57.1%)	0.780
**Smoking** Yes No	37 (35.6%) 83 (43.5%)	67 (64.4%) 108 (56.5%)	0.188
**Physical activity** Yes No	86 (47.5%) 34 (29.8%)	95 (52.5%) 80 (70.2%)	0.003

**Table 4:** Logistic regression analysis of depression and other factors.

**Variables**	**B**	**P-value**	**OR**	**95% Confidence Interval for OR**
**Physical activity** Yes No	- 0.781	0.003	2.18	(1.30, 3.65)
**Gender** Female Male	- 0.597	0.038	0.551	(.313, .969)
**Smoking** Yes No	- -0.611	0.039	0.543	(0.303, 0.971)

## Data Availability

The data and supportive information are available within the article.

## LIST OF ABBREVIATIONS

CESD-R: = Center for Epidemiological Studies Depression Scale Revised
HCW: = Healthcare Workers
SARS-CoV-2: = Severe Acute Respiratory Coronavirus 2
ACSM: = American College of Sports Medicine
IRB: = Institutional Review Board

## References

[1] Chatzittofis A., Karanikola M., Michailidou K., Constantinidou A. (2021). Impact of the COVID-19 Pandemic on the mental health of healthcare workers.. Int. J. Environ. Res. Public Health.

[2] Chew N.W.S., Lee G.K.H., Tan B.Y.Q., Jing M., Goh Y., Ngiam N.J.H., Yeo L.L.L., Ahmad A., Ahmed Khan F., Napolean Shanmugam G., Sharma A.K., Komalkumar R.N., Meenakshi P.V., Shah K., Patel B., Chan B.P.L., Sunny S., Chandra B., Ong J.J.Y., Paliwal P.R., Wong L.Y.H., Sagayanathan R., Chen J.T., Ying Ng A.Y., Teoh H.L., Tsivgoulis G., Ho C.S., Ho R.C., Sharma V.K. (2020). A multinational, multicentre study on the psychological outcomes and associated physical symptoms amongst healthcare workers during COVID-19 outbreak.. Brain Behav. Immun..

[3] Zhang W., Wang K., Yin L., Zhao W., Xue Q., Peng M., Min B., Tian Q., Leng H., Du J., Chang H., Yang Y., Li W., Shangguan F., Yan T., Dong H., Han Y., Wang Y., Cosci F., Wang H. (2020). Mental health and psychosocial problems of medical health workers during the COVID-19 Epidemic in China.. Psychother. Psychosom..

[4] Shanafelt T., Ripp J., Trockel M. (2020). Understanding and addressing sources of anxiety among health care professionals during the COVID-19 pandemic.. JAMA.

[5] Solomou I., Constantinidou F. (2020). Prevalence and predictors of anxiety and depression symptoms during the COVID-19 pandemic and compliance with precautionary measures: Age and sex matter.. Int. J. Environ. Res. Public Health.

[6] Stylianou N., Samouti G., Samoutis G. (2020). Mental health disorders during the COVID-19 outbreak in Cyprus.. J. Med. Life.

[7] Woon LS, Sidi H, Nik Jaafar NR, Leong Bin Abdullah MFI (2020). Mental health status of university healthcare workers during the COVID-19 Pandemic: A post-movement lockdown assessment.. Int J Environ Res Public Health.

[8] Olashore AA, Molebatsi K, Musindo O (2022). Psychosocial predictors of anxiety and depression in a sample of healthcare workers in Botswana during the COVID-19 pandemic: A multicenter cross-sectional study.. SAGE Open Med.

[9] Kandola A., Ashdown-Franks G., Hendrikse J., Sabiston C.M., Stubbs B. (2019). Physical activity and depression: Towards understanding the antidepressant mechanisms of physical activity.. Neurosci. Biobehav. Rev..

[10] World Health Organization (2017). Depression and other common mental disorders: Global health estimates..

[11] Childs E, de Wit H (2014). Regular exercise is associated with emotional resilience to acute stress in healthy adults.. Front Physiol..

[12] Martinsen E.W. (1994). Physical activity and depression: Clinical experience.. Acta Psychiatr. Scand..

[13] Andersson E, Hovland A, Kjellman B, Taube J, Martinsen E (2015). >Fysisk aktivitet lika bra som KBT eller läkemedel vid depression [Physical activity is just as good as CBT or drugs for depression].. Lakartidningen..

[14] Vaquero-Solís M, Tapia-Serrano MA, Hortigüela-Alcalá D, Sierra-Díaz MJ, Sánchez-Miguel PA (2021). Physical activity and quality of life in high school students: Proposals for improving the self-concept in physical education.. Int J Environ Res Public Health..

[15] (2022). Ministry of Health, Amman, Jordan. https://corona.moh.gov.jo/en/MediaCenter/5827.

[16] Al-Amer R.M., Malak M.Z., Aburumman G., Darwish M., Nassar M.S., Darwish M., Randall S. (2019). Prevalence and predictors of depression, anxiety, and stress among Jordanian nurses during the coronavirus disease.. Int J Ment Health Addict.

[17] Alnazly E, Khraisat OM, Al-Bashaireh AM, Bryant CL (2021). Anxiety, depression, stress, fear, and social support during COVID-19 pandemic among Jordanian healthcare workers.. PLoS One.

[18] Al Omari O, Al Sabei S, Al Rawajfah O (2020). Prevalence and predictors of depression, anxiety, and stress among youth at the time of COVID-19: An online cross-sectional multicountry study.. Depress Res Treat..

[19] Basheti IA, Mhaidat QN, Mhaidat HN (2021). Prevalence of anxiety and depression during COVID-19 pandemic among healthcare students in Jordan and its effect on their learning process: A national survey.. PLoS One.

[20] Oteir AO, Nazzal MS, Jaber AF, Alwidyan MT, Raffee LA (2022). Depression, anxiety and insomnia among frontline healthcare workers amid the coronavirus pandemic (COVID-19) in Jordan: A cross-sectional study.. BMJ Open.

[21] Naser A.Y., Dahmash E.Z., Al-Rousan R., Alwafi H., Alrawashdeh H.M., Ghoul I., Abidine A., Bokhary M.A., AL-Hadithi H.T., Ali D., Abuthawabeh R., Abdelwahab G.M., Alhartani Y.J., Al Muhaisen H., Dagash A., Alyami H.S. (2020). Mental health status of the general population, healthcare professionals, and university students during 2019 coronavirus disease outbreak in Jordan: A cross‐sectional study.. Brain Behav..

[22] Bo A., Pouwer F., Juul L., Nicolaisen S.K., Maindal H.T. (2019). Prevalence and correlates of diabetes distress, perceived stress and depressive symptoms among adults with early‐onset Type 2 diabetes: Cross‐sectional survey results from the Danish DD2 study.. Diabet. Med..

[23] Andresen E.M., Malmgren J.A., Carter W.B., Patrick D.L. (1994). Screening for depression in well older adults: Evaluation of a short form of the CES-D (Center for Epidemiologic Studies Depression Scale).. Am. J. Prev. Med..

[24] Zhang W., O’Brien N., Forrest J.I., Salters K.A., Patterson T.L., Montaner J.S.G., Hogg R.S., Lima V.D. (2012). Validating a shortened depression scale (10 item CES-D) among HIV-positive people in British Columbia, Canada.. PLoS One.

[25] Baron E.C., Davies T., Lund C. (2017). Validation of the 10-item Centre for Epidemiological Studies Depression Scale (CES-D-10) in Zulu, Xhosa and Afrikaans populations in South Africa.. BMC Psychiatry.

[26] Kilburn K, Prencipe L, Hjelm L, Peterman A, Handa S, Palermo T (2018). Examination of performance of the Center for Epidemiologic Studies Depression Scale Short Form 10 among African youth in poor, rural households.. BMC Psychiatry.

[27] Fukuti P, Uchôa CLM, Mazzoco MF (2020). How institutions can protect the mental health and psychosocial well-being of their healthcare workers in the current COVID-19 pandemic.. Clinic.

[28] Ingram J., Maciejewski G., Hand C.J. (2020). Changes in diet, sleep, and physical activity are associated with differences in negative mood during COVID-19 lockdown.. Front. Psychol..

[29] Ernstsen L., Havnen A. (2021). Mental health and sleep disturbances in physically active adults during the COVID-19 lockdown in Norway: Does change in physical activity level matter?. Sleep Med..

[30] Chaabane S., Chaabna K., Abraham A., Mamtani R., Cheema S. (2020). Physical activity and sedentary behaviour in the Middle East and North Africa: An overview of systematic reviews and meta-analysis.. Sci. Rep..

[31] Zindah M., Belbeisi A., Walke H., Mokdad A.H. (2008). Obesity and diabetes in Jordan: Findings from the behavioral risk factor surveillance system, 2004.. Prev. Chronic Dis..

[32] Vasile C. (2020). Mental health and immunity.. Exp Ther Med..

[33] Voinov B., Richie W.D., Bailey R.K. (2013). Depression and chronic diseases: It is time for a synergistic mental health and primary care approach. Prim Care Companion CNS Disord..

